# Interim FDG-PET analysis to identify patients with aggressive non-Hodgkin lymphoma who benefit from treatment intensification: a post-hoc analysis of the PETAL trial

**DOI:** 10.1038/s41375-022-01713-y

**Published:** 2022-10-14

**Authors:** Robert Seifert, David Kersting, Christoph Rischpler, Patrick Sandach, Justin Ferdinandus, Wolfgang P. Fendler, Kambiz Rahbar, Matthias Weckesser, Lale Umutlu, Christine Hanoun, Andreas Hüttmann, Hans Christian Reinhardt, Bastian von Tresckow, Ken Herrmann, Ulrich Dührsen, Michael Schäfers

**Affiliations:** 1 Department of Nuclear Medicine, University Hospital Essen, Essen, Germany; 2 Department of Nuclear Medicine, University Hospital Münster, Münster, Germany; 3 German Cancer Consortium (DKTK), University Hospital Essen, Essen, Germany; 4 West German Cancer Center, University Hospital Essen, Essen, Germany; 5 Faculty of Medicine and University Hospital Cologne, Department I of Internal Medicine, Center for Integrated Oncology Aachen Bonn Cologne Duesseldorf, University of Cologne, Cologne, Germany; 6 Department of Diagnostic and Interventional Radiology and Neuroradiology, University Hospital Essen, Essen, Germany; 7 Department of Hematology and Stem Cell Transplantation, University Hospital Essen, University of Duisburg-Essen, Essen, Germany

**Keywords:** Translational research, Cancer imaging

## Abstract

The randomized PETAL trial failed to demonstrate a benefit of interim FDG-PET (iPET)-based treatment intensification over continued standard therapy with CHOP (plus rituximab (R) in CD20-positive lymphomas). We hypothesized that PET analysis of all lymphoma manifestations may identify patients who benefitted from treatment intensification. A previously developed neural network was employed for iPET analysis to identify the highest pathological FDG uptake (max-SUV_AI_) and the mean FDG uptake of all lymphoma manifestations (mean-SUV_AI_). High mean-SUV_AI_ uptake was determined separately for iPET-positive and iPET-negative patients. The endpoint was time-to-progression (TTP). There was a significant interaction of additional rituximab and mean-SUV_AI_ in the iPET-negative group (HR = 0.6, *p* < 0.05). Patients with high mean-SUV_AI_ had significantly prolonged TTP when treated with 6xR-CHOP + 2 R (not reached versus 52 months, *p* < 0.05), whereas max-SUV_manual_ failed to show an impact of additional rituximab. In the iPET-positive group, patients with high mean-SUV_AI_ had a significantly longer TTP with (R-)CHOP than with the Burkitt protocol (14 versus 4 months, *p* < 0.01). Comprehensive iPET evaluation may provide new prognosticators in aggressive lymphoma. Additional application of rituximab was associated with prolonged TTP in iPET-negative patients with high mean-SUV_AI_. Comprehensive iPET interpretation could identify high-risk patients who benefit from study-specific interventions.

## Introduction

Fluorodeoxyglucose positron emission tomography/computed tomography (FDG-PET/CT) performed after 1–4 cycles of chemotherapy (interim PET, iPET) predicts the outcome in aggressive non-Hodgkin lymphoma [1]. In several trials, iPET was employed to identify patients who might benefit from study-specific treatment changes [2, 3]. The ‘Positron Emission Tomography-Guided Therapy of Aggressive Non-Hodgkin Lymphomas’ (PETAL) trial investigated, if treatment intensification can prolong event-free survival (EFS) in patients with a positive (i.e., no sufficient response to previous therapy) iPET result [3]. Treatment intensification in patients with a negative (i.e., response to previous therapy) interim scan was also studied. The study outcome was negative, none of the protocol-mandated treatment changes could improve the outcome. One interpretation of the disappointing result is that the method used to define a positive versus a negative iPET scan may have been inadequate [3].

In clinical routine and in the PETAL study, the assessment of iPET exclusively depends on the lymphoma manifestation with the most intense FDG uptake, which is the basis of the Deauville and delta-SUV_max_ methods [4–6]. Recent studies indicate that more comprehensive FDG-PET/CT analyses taking into account all manifestations of a malignant disease are also feasible [7–9]. However, these analyses are time-consuming, precluding their use in clinical routine. Yet, preliminary data suggest that a neural network based software may assist in the segmentation of lymphoma manifestations [10, 11]. We, therefore, hypothesized that comprehensive FDG-PET analysis is feasible and could be superior to manual single lesion evaluation.

The aim of the present study was twofold. First, using PET data of the PETAL trial, we investigated, if the mean FDG uptake of all lymphoma manifestations prognosticates time-to-progression (TTP). Second, we investigated, if the comprehensive iPET analysis identifies patient subgroups who benefitted from treatment intensification.

## Methods

### Patients

**Fig. 1:**
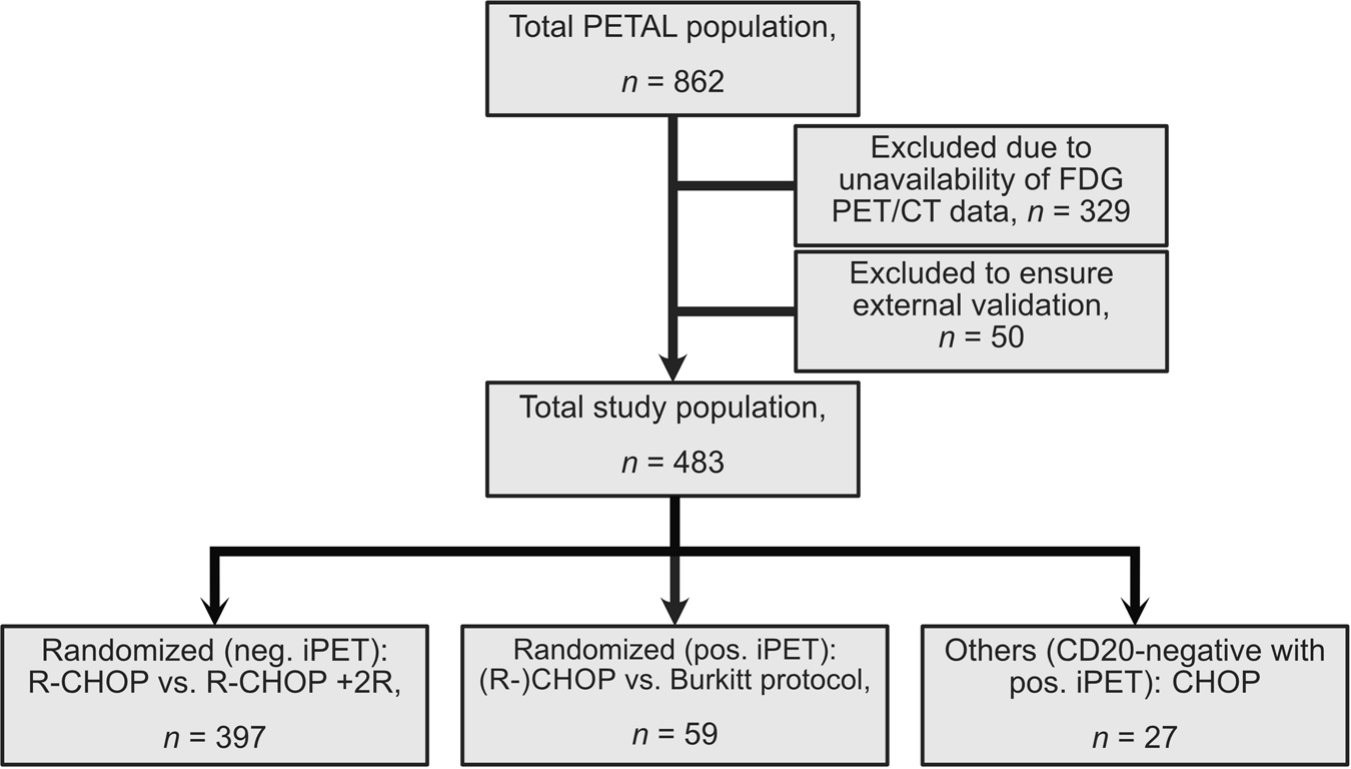
Flow diagram of included patients. Only patients with FDG-PET/CT (no PET-only scans) were included in this analysis. Patients scanned in Münster were excluded, as the AI network used for automated FDG-PET reading was developed and trained in Münster.

Of all patients enrolled in the PETAL trial (*n* = 862), only those with FDG-PET/CT scans available for post-hoc analyses were included in the present study. Additionally, patients scanned at the Department of Nuclear Medicine Münster were excluded from the analysis. This was necessary, as the neural network assisted software used for PET analysis was developed and trained solely with FDG-PET/CT data from Münster, as previously published [12]. Patients gave informed consent for study enrollment. Figure 1 depicts the patients’ flow-chart. Patient characteristics are shown in Table 1. The primary endpoint was TTP defined as time from interim PET until disease progression. The endpoint was defined before reanalysis start and was used to minimize effects of treatment related toxicity or other morbidities.

**Table 1:** Patient characteristics.

Patient characteristics	R-CHOP vs. R-CHOP + 2 R	(R-)CHOP vs. Burkitt protocol*	CHOP*	Total cohort
*n*	397	59	27	483
Age [years]	58 (14.2)	59.1 (13.4)	49.5 (18.1)	57.8 (14.5)
Histological subtype				
DLBCL	312 (78.6%)	35 (59.3%)	1 (3.7%)	348 (72.0%)
Other large B-cell	38 (9.6%)	9 (15.3%)	1 (3.7%)	48 (9.9%)
Lymphoma				
Follicular lymphoma	21 (5.3%)	4 (6.8%)	0 (0%)	25 (5.2%)
T-cell lymphoma	2 (0.5%)	10 (16.9%)	25 (96.2%)	37 (7.7%)
IPI risk group				
Low	158 (39.8%)	14 (23.7%)	13 (48.1%)	185 (38.3%)
Low-intermediate	103 (25.9%)	16 (27.1%)	6 (22.2%)	125 (25.9%)
High-intermediate	85 (21.4%)	16 (27.1%)	4 (14.8%)	105 (21.7%)
High	51 (12.8%)	13 (22.0%)	4 (14.8%)	68 (14.1%)
IPI parameters				
Age > 60	202 (50.9%)	33 (55.9%)	18 (66.7%)	244 (50.5%)
Stage III or IV	223 (56.2%)	44 (74.6%)	17 (63.0%)	284 (58.8%)
Elevated LDH	218 (54.9%)	40 (67.8%)	11 (40.7%)	269 (55.7%)
ECOG > 1	35 (8.8%)	9 (15.3%)	3 (11.1%)	47 (9.7%)
Extranodal site >1	109 (27.5%)	19 (32.2%)	7 (25.9%)	135 (28.0%)
Interim Deauville score >2	279 (70.3%)	55 (93.2%)	20 (74.1%)	354 (73.3%)

Standard deviation is shown in parentheses*.*

*CHOP* cyclophosphamide, doxorubicin, vincristine, prednisone, *DLBCL* diffuse large B-cell lymphoma, *ECOG* Eastern Cooperative Oncology Group performance status, *IPI* International Prognostic Index, *LDH* lactate dehydrogenase, *R* rituximab.

*Interim PET-positive patients with CD20-negative lymphomas received CHOP or CHOP followed by the Burkitt protocol without rituximab. Interim PET-negative patients with CD20-negative lymphomas received CHOP without rituximab.

### Randomization

**Fig. 2:**
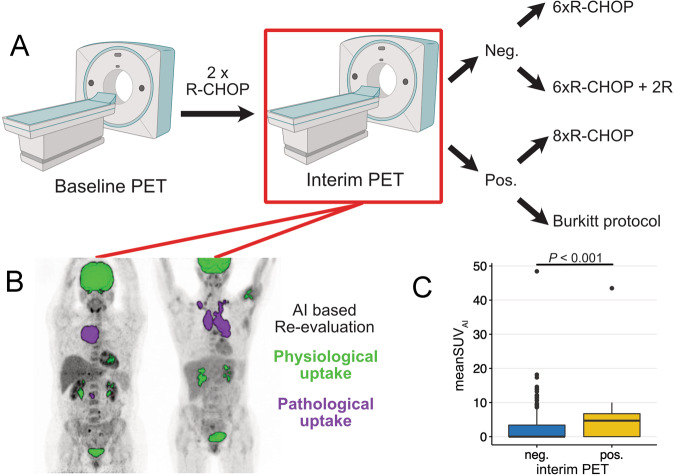
Overall workflow of the PETAL trial and the AI-based re-evaluation. In case of a negative interim PET result, patients were randomized to receive either 6xR-CHOP or 6xR-CHOP plus two additional rituximab administrations. A negative interim PET scan was defined as an SUV decrease >66% of the most FDG-avid lesion compared to the baseline scan. Patients with a positive interim scan were randomized to receive either 8x(R-)CHOP or 2x(R-)CHOP followed by 6 blocks of the Burkitt protocol (R was restricted to CD20-positive lymphomas) (**A**). The interim PET scans were re-evaluated using an AI-based software to automatically quantify FDG uptake (**B**). Patients with a positive interim PET had statistically significantly higher mean-SUV_AI_ compared to those with a negative interim PET (**C**).

Randomization was based on the iPET evaluation using the delta-SUV_max_ method. Patients with a decline of FDG uptake >66% in the most FDG-avid lesion compared to the baseline scan were regarded to have a negative interim PET scan; patients with a lesser decline, an increase, or new lesions were considered to have a positive scan (Fig. 2).

Patients with CD20-positive lymphomas and a negative interim PET scan were (pseudo-)randomized to receive a total of 6 cycles of R-CHOP (rituximab, cyclophosphamide, doxorubicin, vincristine, prednisone; 2 cycles before and 4 cycles after iPET scanning) or 6xR-CHOP plus 2 additional applications of rituximab. As detailed in a previous secondary analysis of the PETAL trial, pseudo-randomization was due to the fact that all patients treated with 6xR-CHOP or 6xR-CHOP + 2 R were included, and not only those specifically randomized between these options [13]. Before the trial period of randomization, all iPET-negative patients with CD20-positive lymphomas received 6xR-CHOP, and after the conclusion of randomization, all such patients received 6xR-CHOP + 2 R.

Patients with a positive iPET scan were randomized to receive a total of 8 cycles of (R-)CHOP or 2 cycles of (R-)CHOP followed by 6 blocks of an intensive Burkitt’s lymphoma protocol. Rituximab was restricted to patients with CD20-positive lymphomas.

### Image analysis

The manual image analysis of the PETAL trial was used for comparison. For re-analysis, the neural network-based PET-assisted reporting system software prototype (PARS) was employed (Siemens Healthineers, Knoxville, TN, USA). The tool is distributed by Siemens upon request. The AUC of this fully automated neural network used for the segmentation of all lymphoma manifestations is 0.95 (95% confidence interval CI: 0.92–0.97), as previously published [12]. PARS identifies all FDG-PET foci with elevated uptake and rates each as either pathological or physiological, using the pretrained networks (Fig. 2). All lymphoma manifestations can thus be segmented fully automatically.

### Image metrics

In the original PETAL publication, the SUV_max_ of the single most FDG-avid lesion was used to assess the overall metabolic activity of the lymphoma [3]. This manually determined interim FDG-PET value is designated max-SUV_manual_ in this study.

Using the PARS neural network (AI), the lymphoma lesion with the highest FDG avidity was measured and designated max-SUV_AI_. Additionally, PARS automatically segmented all lymphoma manifestations in the acquired PET images. We argued that averaging the FDG uptake of each lesion may lead to an improved assessment of the disease severity compared to the uptake of only the hottest lesion. Therefore, the metric mean-SUV_AI_ was introduced, which denotes the average SUV of all segmented lesions. The three metrics (max-SUV_manual_, max-SUV_AI_, mean-SUV_AI_) were tested as PET-derived prognosticators.

### Statistical analysis

The R language and environment were used for Pearson correlation, log-rank test, uni- and multivariable Cox regression analysis and descriptive statistics [14]. The STARD and TRIPOD guidelines were followed. For the interaction analysis in Cox regression, the treatment was coded as a dummy variable with the range [0–1], the intensified treatment (6xR-CHOP + 2 R or the Burkitt protocol, respectively) being coded as 1. To determine high and low PET uptake, the log-rank statistic of the treatment difference between standard and intensified arm was maximized; this was done separately for the group of patients with a positive or a negative iPET. PET-derived parameters were log-transformed due to skewed distributions prior to Cox regression analysis. Mean liver uptake was added before the analysis. The H_0_ hypotheses (treatment intensification does not prolong TTP) were rejected if the *p* value was <0.05.

## Results

### Patient characteristics

**Fig. 3:**
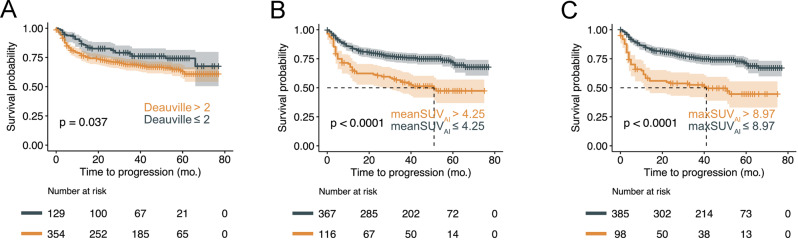
Deauville score versus mean-SUV_AI_ and max-SUV_AI_ for TTP stratification. All included patients were stratified by the Deauville response (**A**) and the cut-off maximizing the survival difference of mean-SUV_AI_ (**B**) and max-SUV_AI_ (**C**). Patients with a Deauville score greater than 2 had statistically significantly shorter TTP. Likewise, patients with a mean-SUV_AI_ or a max-SUV_AI_ greater than the cut-off had statistically significantly shorter TTP.

A total of 483 patients were included in this secondary analysis of the PETAL study of whom 397 were (pseudo-)randomized to 6xR-CHOP versus 6xR-CHOP + 2 R. A positive iPET was observed in 59 patients who were randomized to receive either 8x(R-)CHOP or 2x(R-)CHOP followed by the Burkitt protocol. Detailed patient characteristics are shown in Table 1 and Supplementary Table 1. Figure 3 shows TTP of the cohort dichotomized by the Deauville scale, the mean-SUV_AI_ or the max-SUV_AI_.

### Prognostication of TTP in the total cohort

Max-SUV_manual_ was a statistically significant prognosticator of TTP in univariable Cox regression analysis (hazard ratio [HR] 4.109, 95% CI: 2.922–5.780, *p* < 0.001). The same was true for max-SUV_AI_ (HR 1.334, 95% CI: 1.185–1.501, *p* < 0.001) and mean-SUV_AI_ (HR 1.430, 95% CI: 1.224–1.670, *p* < 0.001). Multivariable Cox regression analysis of mean-SUV_AI_ adjusted for the parameters of the International Prognostic Index (IPI) is shown in Supplementary Table 2 (HR 1.572, 95% CI: 1.294–1.909, *p* < 0.001).

### Effect of therapy intensification in the randomized cohorts

**Fig. 4:**
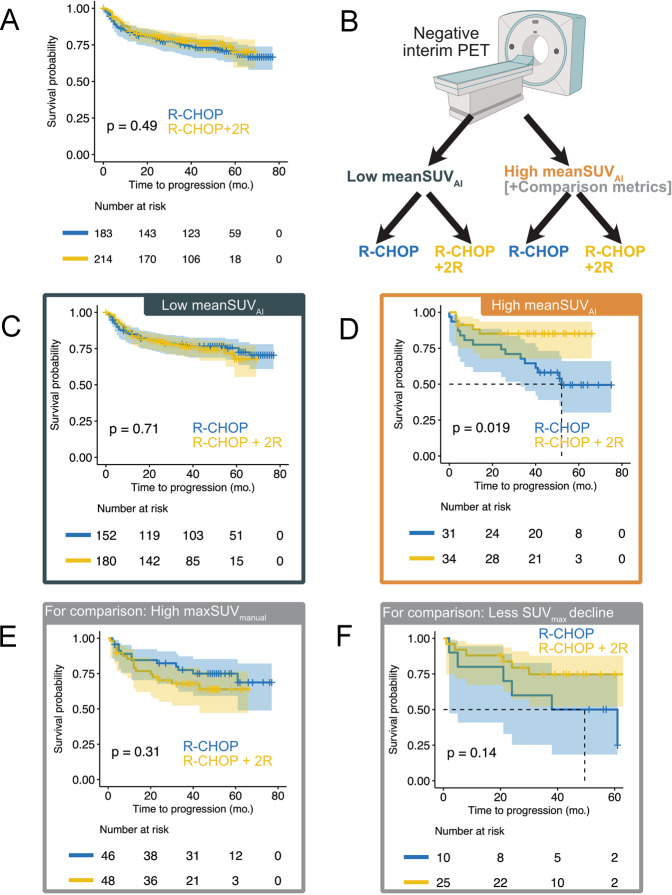
Effect of additional rituximab in patients with a negative interim PET scan. Overall, patients treated with two additional cycles of rituximab did not show a significantly prolonged TTP (**A**). An optimized mean-SUV_AI_ threshold (SUV 4.89) was used to classify patients with a negative iPET into those with high versus low FDG uptake (**B**). In patients with low mean-SUV_AI_, TTP was not affected by additional rituximab (**C**). In contrast, patients with high mean-SUV_AI_ receiving additional rituximab had significantly prolonged TTP (**D**). For comparison, the conventional metrics max-SUV_manual_ (**E**) and decline in SUV_max_ between baseline and interim PET (**F**) could not identify patients who had a TTP benefit in response to treatment with additional rituximab.

**Fig. 5:**
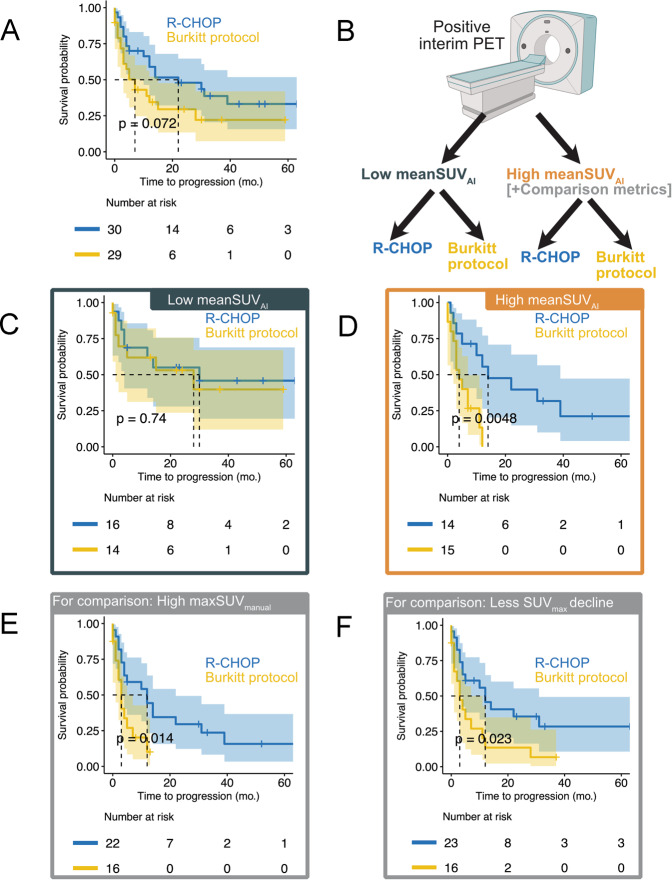
Effect of treatment intensification by the Burkitt protocol in patients with a positive interim PET scan. Overall, patients receiving treatment intensification by the Burkitt protocol did not have a statistically longer TTP than patients receiving standard R-CHOP (**A**). Patients with a positive interim PET scan were grouped by an optimized mean-SUV_AI_ threshold (SUV 4.78) into those with high versus low FDG uptake (**B**). In patients with low mean-SUV_AI_, no statistically significant difference between patients treated with R-CHOP or the Burkitt protocol was observed (**C**). In contrast, in the high mean-SUV_AI_ subgroup, patients treated with R-CHOP had statistically significantly longer TTP than those treated with the Burkitt protocol (**D**). For comparison, the conventional metrics max-SUV_manual_ (**E**) and decline in SUV_max_ between baseline and interim PET (**F**) could also identify patients who showed longer TTP in response to treatment with R-CHOP over the Burkitt protocol.

In the interim PET-negative group, there was no statistically significant difference in TTP between patients treated with 6xR-CHOP or 6xR-CHOP + 2 R (medians not reached; HR 0.872, 95% CI: 0.590–1.288, *p* > 0.05; Fig. 4A). Likewise, in the interim PET-positive group, there was no statistically significant difference in TTP between patients treated with 8x(R-)CHOP or 2x(R-)CHOP followed by the Burkitt protocol (7 versus 22 months; HR 1.798, 95% CI: 0.940–3.441, *p* > 0.05, Fig. 5A).

### Identification of patients in the iPET-negative subgroup who benefitted from treatment intensification by additional rituximab

All patients referred to 6xR-CHOP or 6xR-CHOP + 2 R were considered in this analysis. To investigate a potential interaction of treatment regime (coded: 6xR-CHOP[0]; 6xR-CHOP + 2 R(1)) and interim PET parameter (as a continuous variable), interaction terms were evaluated in multivariable Cox regressions. A significant interaction term would indicate that patients with higher uptake benefit from intensified treatment. The interaction term (treatment regime × max-SUV_manual_) was not a statistically significant prognosticator of TTP (HR 1.339, 95% CI: 0.334–5.371, *p* > 0.05) in an analysis including treatment regime (6xR-CHOP or 6xR-CHOP + 2 R), max-SUV_manual_, and the IPI score. The same was true for the interaction term employing max-SUV_AI_ (HR 0.745, 95% CI: 0.532–1.045, *p* > 0.05). In contrast, an interaction term employing mean-SUV_AI_ (treatment regime × mean-SUV_AI_) was a statistically significant prognosticator of TTP (HR 0.579, 95% CI: 0.340–0.987, *p* < 0.05) (Supplementary Table 3).

The optimal mean-SUV_AI_ threshold for patient stratification (SUV 4.89) in the interim PET-negative subgroup was used to group patients into high versus low PET uptake (Fig. 4). In patients with high mean-SUV_AI_, those treated with 6xR-CHOP + 2 R had significantly longer TTP than those treated with 6xR-CHOP alone (median not reached versus 52 months; HR 0.316, 95% CI: 0.114–0.875, *p* < 0.05). This was not true for the conventional metrics max-SUV_manual_ or the decrease in SUV_max_ between baseline and interim PET (Fig. 4); no subgroup who benefitted from treatment intervention could be identified with these metrics. In the low mean-SUV_AI_ group, no statistically significant difference was observed between 6xR-CHOP and 6xR-CHOP + 2 R (medians not reached; HR 1.086, 95% CI: 0.702–1.680, *p* > 0.05).

### Identification of patients in the iPET-positive subgroup who had a disadvantage from treatment intensification by the Burkitt protocol

The interaction term (treatment regime × mean-SUV_AI_) was not a statistically significant prognosticator of TTP (HR 0.401, 95% CI: 0.157–1.023, *p* > 0.05) in a multivariable Cox regression analysis adjusted for treatment regime (8x(R-)CHOP versus 2x(R-)CHOP followed by the Burkitt protocol), mean-SUV_AI_, and the IPI score (coded: (R-)CHOP[0]; Burkitt protocol(1); Supplementary Table 4). However, mean-SUV_AI_ was a statistically significant prognosticator of TTP (HR 2.333, 95% CI: 2.091–4.989, *p* < 0.05).

The optimal mean-SUV_AI_ threshold (SUV 4.78) for patient stratification in the interim PET-positive subgroup was used to group patients into those with high versus low uptake. All patients who were randomly assigned to 8x(R-)CHOP or 2x(R-)CHOP followed by the Burkitt protocol were evaluated in this analysis. In the high mean-SUV_AI_ group, patients treated with the Burkitt protocol showed significantly shorter TTP than patients continuing on (R-)CHOP (4 versus 14 months; HR 4.104, 95% CI: 1.455–11.580, *p* < 0.01; Fig. 5). For comparison, the conventional PET metrics max-SUV_manual_ and the decrease in SUV_max_ between baseline and interim PET could also identify subgroups of patients who were disadvantaged from the Burkitt protocol (Fig. 5). In patients with low mean-SUV_AI_, no statistically significant difference in TTP was observed between the treatment arms (28 versus 30 months; HR 1.909, 95% CI: 0.43–3.293, *p* > 0.05).

## Discussion

The interim FDG-PET scans of the treatment intensification PETAL trial were re-analyzed in a comprehensive PET analysis to segment all lymphoma manifestations. The following principal findings arise from this analysis: (1) A fully automated analysis of interim FDG-PET/CTs from lymphoma patients is feasible. (2) The biomarkers derived from the comprehensive PET analysis are statistically significant prognosticators of TTP. (3) The mean-SUV_AI_ parameter identified patients that benefitted from additional application of rituximab as treatment intensification, which could not be achieved using conventional PET metrics.

(R-)CHOP is the standard first-line treatment for patients with aggressive lymphoma, with cure rates of 60–70%. In patients with (multiply) relapsed disease, several treatment options exist, such as high-dose chemotherapy with autologous hematopoietic stem cell transplantation, allogeneic transplantation, CAR-T cell therapy, immunomodulation, and others [15–17]. Current methods for early prediction of treatment failure, including Deauville-based iPET assessment, appear insufficient.

FDG-PET has a long track record of monitoring initial treatment response to systemic anti-cancer therapy [2, 3, 18]. In principle, early detection of treatment failure could trigger a change in therapy, aiming at improved outcome. However, often only a single target lesion is used to assess treatment failure and guide subsequent therapies. A single lesion, however, cannot accurately capture disease extent and severity. To overcome this limitation, a recent approach tries to employ ctDNA levels as comprehensive biomarker to assess the total lymphoma burden [19]. However, in FDG-PET is comprehensively analyzed, it can also quantify the total lymphoma burden and assess the metabolic heterogeneity of all manifestations. As the delineation of all disease manifestations is too time-consuming for clinical routine, AI-based PET analysis software, like the PARS prototype and others, have been proposed [12, 20].

For the conventional metric max-SUV_manual_, which takes account of a single lymphoma manifestation, no statistically significant interaction of treatment intensification by additional rituximab was found in the present analysis. In contrast, for the mean-SUV_AI_ metric, which averages the FDG uptake of all lymphoma manifestations, a statistically significant interaction with treatment intensification was observed. This indicates that the benefit of treatment intensification through additional rituximab is growing with increasing mean-SUV_AI_. This was corroborated by looking at patients with high mean-SUV_AI_ who had statistically significantly longer survival when treated with two additional rituximab doses than with 6xR-CHOP alone. Interestingly, patients with high mean-SUV_AI_ had higher baseline SUV_max_ compared to patients with low mean-SUV_AI_ (Supplementary Table 5). This indicates that patients with high mean-SUV_AI_ might erroneously be read as iPET-negative due to their high baseline SUV_max_, which could lead to a more pronounced relative reduction, despite metabolically active residual lymphoma at the interim timepoint. The finding is in line with recent studies indicating that a more complex PET analysis of lymphoma patients is superior to the IPI index [21].

In patients randomized to 8x(R-)CHOP versus 2x(R-)CHOP followed by the Burkitt protocol, no statistically significant interaction of a PET parameter and treatment intensification was found. However, patients with high mean-SUV_AI_ had statistically significantly longer TTP when they were not treated with the Burkitt protocol. This seems paradoxical as especially patients with very high residual tumor activity seemed to have a disadvantage from therapy intensification. Also, conventional PET metrics such as highest uptake or change in highest uptake between baseline and interim could identify patients who were disadvantaged by the Burkitt protocol; highlighting the need for comprehensive PET analysis. The data, however, need to be interpreted with caution because of imbalances in baseline characteristics (Supplementary Table 1).

Our study has several limitations. First, it was a retrospective re-analysis of the prospective PETAL trial. The present analysis was not pre-planned, which might cause an observational bias. Additionally, all patients receiving 6xR-CHOP and 6xR-CHOP + 2 R were included, but only a subfraction was truly randomized (178 of 397 patients). However, non-randomized patients receiving 6xR-CHOP or 6xR-CHOP + 2 R were recruited using the same inclusion criteria in the beginning and at the end of the study period, respectively, which should minimize potential biases. Finally, our primary endpoint was TTP which best reflects the impact of therapy on outcome [7–9]. In contrast, the PETAL trial employed event-free survival (EFS), which also included death unrelated to lymphoma and events such as treatment-related toxicity.

## Conclusion

A comprehensive analysis of interim FDG-PET in patients with aggressive non-Hodgkin lymphoma is feasible. In the PETAL trial, this novel approach identified patients who benefitted from protocol-mandated treatment intensification. This might indicate the superiority of average FDG avidity over conventional metrics restricted to the metabolically most active lesion. Future studies should evaluate the use of automated image analysis for interim PET assessment to identify patients who may benefit from a change in therapy.

## Supplementary information

Supplement

## Data Availability

Data supporting the presented findings may be shared upon request. No consent for sharing of image data was given. The PARS prototype software is available through Siemens Healthineers.
