# Supplementary material for: Interim FDG-PET analysis to identify patients with aggressive non-Hodgkin lymphoma who benefit from treatment intensification: a post-hoc analysis of the PETAL trial
Source: Leukemia. 2022 Oct 14;36(12):2845–52. doi: 10.1038/s41375-022-01713-y (PMC9712103; doi:10.1038/s41375-022-01713-y)
Supplement: Supplementary file 1 — Supplement [file 41375_2022_1713_MOESM1_ESM.docx]

## Supplemental Table 1 – Patient characteristics shown separately for the (pseudo-)randomized treatment groups

| **Patient characteristics** | **6xR-CHOP** | **6xR-CHOP +2R** | **8x(R-)CHOP** | **2x(R-)CHOP +Burkitt** |
| --- | --- | --- | --- | --- |
| n | 183 | 214 | 30 | 29 |
| Age [years] | 58.8 | 57.7 (13.4) | 58.4 (13.7) | 59.8 (13.3) |
| Histological subtype |  |  |  |  |
| DLBCL | 142 (77.6%) | 170 (79.4%) | 21 (70.0%) | 14 (48.3%) |
| Other large B-cell lymphoma | 20 (10.9%) | 18 (18.4%) | 4 (13.3%) | 5 (17.2%) |
| Follicular lymphoma | 9 (4.9%) | 12 (5.6%) | 1 (3.3%) | 3 (10.3%) |
| T-cell lymphoma | 1 (0.5%) | 1 (0.5%) | 3 (10.0%) | 7 (24.1%) |
| IPI risk group |  |  |  |  |
| Low | 68 (26.8%) | 90 (42.1%) | 7 (23.3%) | 7 (24.1%) |
| Low-intermediate | 49 (26.8%) | 54 (25.2%) | 9 (30.0%) | 7 (24.1%) |
| High-intermediate | 39 (21.3%) | 46 (21.5%) | 8 (26.7%) | 8 (27.6%) |
| High | 27 (14.8%) | 24 (11.2%) | 6 (20.0%) | 7 (24.1%) |
| IPI parameters |  |  |  |  |
| Age > 60 | 104 (56.8%) | 99 (46.3%) | 17 (56.7%) | 16 (55.2%) |
| Stage III or IV | 131 (71.6%) | 119 (55.6%) | 21 (70.0%) | 23 (79.3%) |
| Elevated LDH | 101 (55.2%) | 117 (54.7%) | 18 (60.0%) | 22 (75.9%) |
| ECOG >1 | 16 (8.7%) | 19 (8.9%) | 4 (13.3%) | 5 (17.2%) |
| Extranodal site >1 | 54 (29.5%) | 55 (25.7%) | 11 (36.7%) | 8 (27.6%) |
| Interim Deauville score >2 | 131 (71.6%) | 149 (69.2%) | 28 (93.9%) | 27 (93.1%) |
| Standard deviation is shown in parentheses. Abbreviations: CHOP, cyclophosphamide, doxorubicin, vincristine, prednisone; DLBCL, diffuse large B-cell lymphoma; ECOG, Eastern Cooperative Oncology Group performance status; IPI, International Prognostic Index; LDH, lactate dehydrogenase; R, rituximab.  Interim PET-negative patients received either 6xR-CHOP or 6xR-CHOP+2R. Interim-PET-positive patients received either 8x(R-)CHOP or 2x(R-)CHOP followed by the Burkitt protocol. R was restricted to CD20-positive lymphomas. | | | | |

## Supplemental Table 2 – Prognostication of time-to-progression in the total cohort by multivariable Cox regression analysis

## Supplemental Table 3 – Interaction of treatment regime and mean-SUV_AI_ in interim PET-negative patients with CD20-positive lymphomas (pseudo-)randomized to 6xR-CHOP versus 6xR-CHOP+2R for prognostication of time-to-progression in multivariable Cox regression

## Supplemental Table 4 – Multivariable Cox regression analysis for prognostication of time-to-progression in interim PET-positive patients randomized to 8x(R-)CHOP versus 2x(R‑)CHOP followed by the Burkitt protocol

## Supplemental Table 5 – Baseline and interim FDG uptake in PETs with respect to high and low mean-SUV_AI_

|  | **Low mean-SUV_AI_ group** | **High mean-SUV_AI_ group** | | | ***P* value** |
| --- | --- | --- | --- | --- | --- |
| Baseline SUV_max_ | 20.39 | | 24.62 | 0.0067 | |
| Interim SUV_max_  (= max-SUV_manual_) | 3.26 | | 4.20 | 0.0032 | |
